# Urethrotomy

**Published:** 1874-06

**Authors:** Fessenden N. Otis


*NEW YORK MEDICAL JOURNAL—April,* 1874:

URETHROTOMY—*By Fessenden N. Otis, M.D.*—In closing this somewhat desultory paper, I would like to be distinctly understood as claiming that stricture, as ordinarily met with, is *absolutely within the reach of curative measures;* that, if completely divided, and this division maintained by suitable means until healing of the parts has occurred, no *recontraction* can ever take place; that dilatation, temporary or permanent, *is never more than a palliative measure,* unless carried to a point sufficient to *completely rupture the stricture*; that the division of stricture is not more hazardous, to say the least, than permanent dilatation—that is, by introduction of dilating instruments, which are required to remain *in situ* for hours or days—or than rapid dilatation, which requires instruments of increasing sizes to be introduced at one sitting; and, I may venture to say, scarcely more likely to produce trouble than temporary or transient dilatation, as usually practiced by surgeons, which is simply to pass a sound or bougie gently through the urethra, and to be immediately withdrawn; the same to be reintroduced, at intervals of two or three days or weeks, for the rest of the natural life of the unfortunate patient.

